# Incidence rate of metastases in the oral cavity: a review of all metastatic lesions in the oral cavity

**DOI:** 10.4317/medoral.24625

**Published:** 2021-05-23

**Authors:** Clàudia Oliver-Puigdomènech, Beatriz González-Navarro, Carlos Polis-Yanes, Albert Estrugo-Devesa, Enric Jané-Salas, José López-López

**Affiliations:** 1 School of Dentistry, University Campus of Bellvitge, University of Barcelona, Spain; 2 Department of Odontostomatology, Faculty of Medicine and Health Sciences (Dentistry), Odontological Hospital University of Barcelona, Spain; 3 Oral Health and Masticatory System Group (Bellvitge Biomedical Research Institute) IDIBELL, University of Barcelona, Spain; 4 Facultative Director and Responsible of the Medical and Surgical Area, Barcelona University Dental Hospital (HOUB), Spain

## Abstract

**Background:**

Metastases in the oral cavity are rare and account for only 1 to 3% of all malignant lesions in this area. The primary location from which most metastases have been described in the oral cavity in adult patients include lungs, breasts, kidneys and colon.

**Material and Methods:**

A systematic search of the literature was carried out following the PRISMA statement in PubMed database. Clinical trials and case series published in the last 10 years [2010-2020] were eligible to be selected. The headings and keywords used in the searches were “cancer” AND “oral metastases”, “incidence” AND “oral metastases”, “oral metastases” AND “jaw bone”, “oral metastases” AND “soft tissue”.

**Results:**

For the study of the incidence of metastases in the oral cavity, 9 reports of clinical trials and 7 retrospective studies of case series have been included in this article. The primary locations from which more metastases have been described in the oral cavity are lungs (30.6% or 183 cases), breasts (22.2% or 133 cases), liver (15.5% or 93 cases), prostate (9 % or 54 cases), thyroid glands (8.1% or 49 cases), kidneys (7.3% or 44 cases), skin (2.3% or 14 cases), soft tissues (2% or 12 cases), colon (2% or 12 cases) and gastrointestinal (0.6% or 4 cases). These metastases have a predilection for hard tissues. The clinical presentation of these lesions varies from painless granulomatous lesions to lytic areas in the jaws.

**Conclusions:**

Although metastases in the oral cavity is an uncommon pathology, early diagnosis is needed so that in the event that it is the first manifestation, it allows the primary tumor to be diagnosed as soon as possible.

** Key words:**Cancer, oral metastases, incidence, jaw bone, soft tissue.

## Introduction

Oral metastases is known to be a cancerous pathology that has its origin in another primary location from which it has spread. Tumors that metastasize in the oral region can, in many cases, be the secondary spread of another metastasis (1).

Metastases in the oral cavity are rare and account for only 1 to 3% of all malignant lesions in this area (1,2). These metastases have greater predilection for the jaws than for soft tissues and this has been reported in the literature (3). Specifically, of the maxillary bones, the mandible is the most frequently affected location. In soft tissues, the attached gingiva is the most common location for the settlement of metastases, followed by the tongue (4,5). It is believed that the dissemination mechanism of a neoplasm from the primary location to the oral cavity is basically through blood dissemination, through the epidural venous plexus (Batson venous system), which vascularizes the axial skeleton and the head region and neck (6-8).

The primary location from which more metastases have been described in the oral cavity in adult patients includes lungs, breasts, kidneys and colon (1). Differences are found based on gender: for men, the most commonly reported location are the lungs, followed by prostate, liver and kidneys. On the other hand, in women, breasts are the primary location most associated with oral metastases, followed by adrenal glands, tumors in the genital organs and colorectal organs (6,9).

Metastases treatment will vary depending on the primary tumor type and its spread degree, the patient overall health and tumor location (10). Management includes surgical resection, sometimes combined with radio or chemotherapy (3). In healthy patients who respond properly to primary tumor therapy, aggressive treatment is recommended for oral metastases. However, if the primary tumor is recurrent and has other metastases, treatment of the oral lesion should be minimally invasive (11,12).

This type of metastases is a sign of widespread primary cancer and is a sign of poor prognosis, with 5-year survival rate below 5% (3,4,6,7).

In approximately 30% of patients with metastases in the jaw, the location of the primary tumor is unknown (10,12). This is the reason why knowing how to identify malignant lesions in the oral cavity is of great value, taking them into account in the differential diagnosis of inflammatory and reactive lesions, which are more common in this region. These lesions will require a biopsy, which will confirm that it is a spread secondary lesion of another neoplasm coming from another organ.

This review aims to update the knowledge of the incidence rate of metastases in the oral cavity and the primary tumors that cause them.

## Material and Methods

A systematic search of the literature was carried out following the PRISMA statement (“Preferred Reporting Items for Systematic Reviews and Meta-Analyzes”) in order to collect the greatest scientific evidence regarding the incidence of oral cavity metastases (13).

The search strategy consisted of carrying out different bibliographic searches in the PubMed database without applying filters. The headings and keywords used in the searches were “cancer” AND “oral metastases”, “incidence” AND “oral metastases”, “oral metastases” AND “jaw bone”, “oral metastases” AND “soft tissue”.

Those articles performed in humans that are clinical cases and case series, published in the last 10 years (2010 to 2020 period) were eligible to be selected. Studies published more than 10 years ago, that are not in Spanish or English, that are neither clinical cases nor clinical case series, as well as articles in which metastases does not occur in the oral cavity were excluded from this review.

## Results

**Figure 1:**
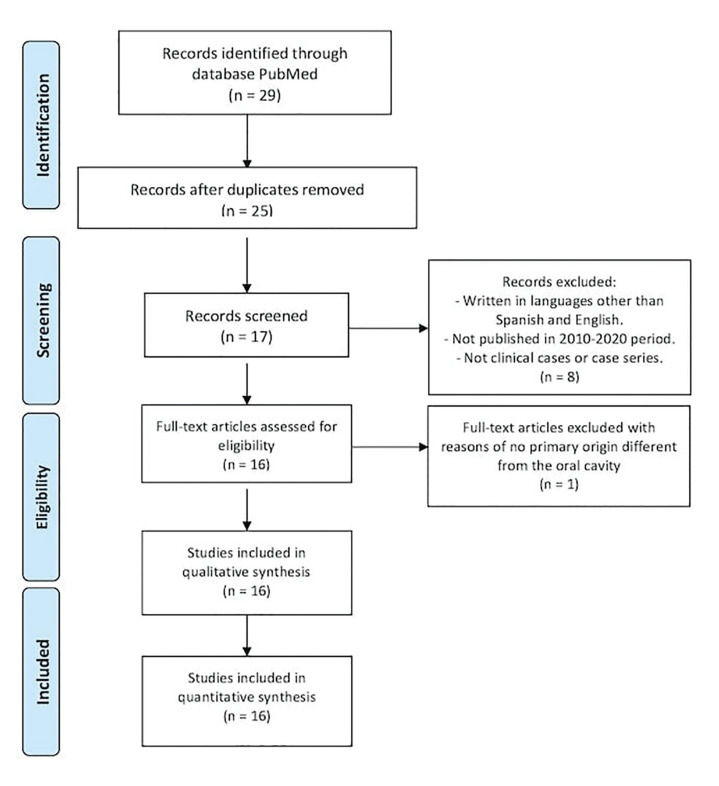
PRISMA flow chart.

Fig. 1 flow chart exhibits the results of the initial literature search in PubMed. After discarding duplicate articles, 25 articles are left, of which we discarded 9 because they do not meet the inclusion criteria.

Finally, for the study of the incidence rate of metastases in the oral cavity, 9 clinical cases (5,7,8,14-19) and 7 retrospective studies of case series have been included in this article (Table 1) (2,4,12,20-23). From 609 tumors analyzed, 236 are in women (38.7%) and 373 are in men (61.2%), with a female/male distribution ratio of 0.63:1. In the clinical cases and case series studied, the mean age at the time of the oral metastases diagnosis is 62.1 years (2,4,5,7,8,12,14-19,21-24).

From a geographical point of view, the cases included in the study are based in the first place on European populations such as the Italian, Croatian or French with 10 case reports (2,5,7,8,12,14,17,20,24) followed by the United States (USA) with 2 cases (15,20) and finally countries such as Korea (21), Israel (4), India (17) and Brazil (18) of which only a single clinical case or case series has been included.

There are many possible locations of primary tumors but in our review the most reported are lungs (30.6% or 183 cases), followed by breasts neoplasms (22.2% or 133 cases), liver (15.5 % or 93 cases), prostate (9% or 54 cases), thyroid glands (8.1% or 49 cases), kidney (7.3% or 44 cases), skin (2.3% or 14 cases), soft tissues (2% or 12 cases), colon (2% or 12 cases), gastrointestinal (0.6% or 4 cases). The tumors of which only 1 case has been reported in total in our review are rectum, esophagus, cervix, testicles and unknown origin (2,4,5,7,8,12,14-19,21-24).

Regarding metastases in the oral cavity, they can occur in both hard and soft tissues, sometimes affecting both at the same time (Table 2). In this review, we demonstrated greater hard tissue involvement in women and greater soft tissue involvement in men. In addition, we can affirm that, in women, when the primary tumor is located in breasts, there is a greater involvement of the maxillary bones compared to soft tissues. On the other hand, if the primary tumor is located in kidneys, lung, skin, or colorectal, the ones most affected tissues by metastases are the soft tissues. Regarding male gender, proportions are different, being tumors such as lung, kidney, skin and soft tissues the ones that most metastasize in soft tissues of the oral cavity. In contrast, tumors located in organs of the digestive system, liver or prostate, metastasize more often in hard tissues (2,4,5,7,8,12,14-19,21-24). Data collected from articles that meet the inclusion criteria except for the articles by Irani (22) and Liu *et al*. (20) have been included in Table 2. These two have not been included because they do not specify the data that is compared in the Table 2. Thus, Table 2 shows data from 138 cases of primary tumors that produce metastases in the oral cavity, either in hard tissues, in soft tissues or in both (2,4,5,7,8,12,14-19,21-24).

In order of frequency, the histopathological type of tumors is: adenocarcinoma, squamous carcinoma, melanoma and sarcoma (2,4,5,7,8,12,14-19,21-24).

Prognosis of patients with metastases in the oral cavity is poor, with a mortality rate in this study close to 70%. Death occurs in a period of time ranging from 3 weeks after the diagnosis of metastases to 30 months, with a mean death at 9.81 months (2,4,5,7,8,12,14-19,21-24).

**Table 1:**
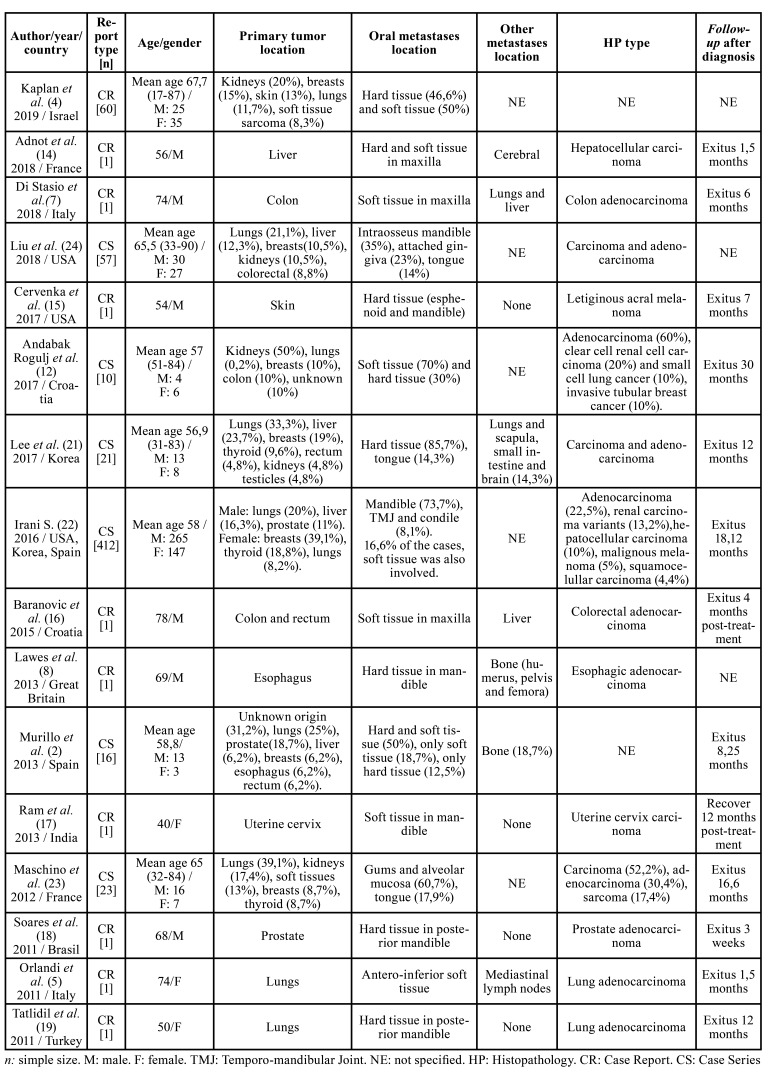
Case reports and case series included in this review.

**Table 2:**
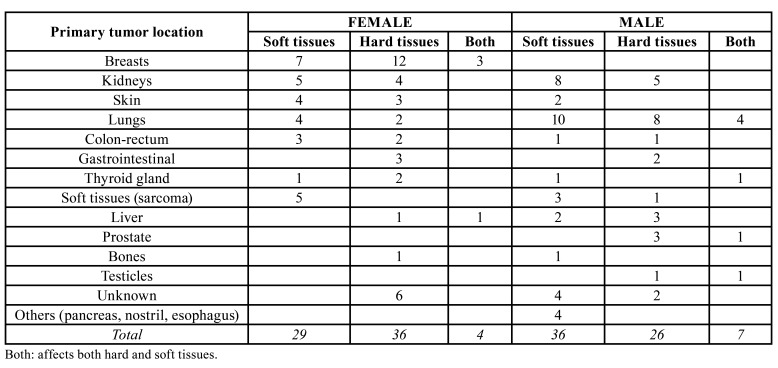
Distribution of the metastases in the oral cavity according to gender.

## Discussion

Tumor metastases in the oral cavity are uncommon, occurring in 1-3% of cases (1,22). They usually appear as lesions in hard tissues of the maxilla and mandible, being the mandible the most frequent location (3,8,15,21). They can be lesions that originate in soft tissues and end up infiltrating bone or lesions that initially appear in hard tissues and end up resulting on soft tissues of the oral cavity (5,15,17,19).

Most of the patients diagnosed with metastases in the oral cavity are between the ages of 50 and 70 years old, although the age range can vary from 20 to 90 years old (1,4). Patients with metastases in the maxilla are usually younger than patients with soft tissue metastases, a result that is consistent with previous studies (6).

Predilection of metastases in the oral region for a particular gender is still considered controversial. According to the retrospective study by Lee *et al*. (21) in 21 patients diagnosed with oral metastases, the female/male ratio was 1:1.63, showing a predominance of the male gender. Same ratio occurs in the case series by Maschino *et al*. (23) where the ratio is 1:2.3 in favor of men. In our review, the female/male ratio is 0.63:1, so our data matches the majority of published literature regarding distribution by gender. On the other hand, in the case series published by Kaplan *et al*. (4), this ratio is 1.4:1, including 35 women and 25 men among their 60 cases studied.

According to what has been reported, we could affirm that gender ratio is almost 1:1 when speaking of metastases in the oral cavity, although location of metastases in the oral region differs when we refer to men or women (25). Hirshberg *et al*. (6) reported in 2008 that metastases in jaws have a predilection for females. However, according to another analysis of 390 cases, a predilection for the male sex was observed, both in metastases in the oral mucosa and those that occur in jaws (9). These patterns probably differ depending on factors such as race and age of the studied population, as well as the way to study these populations in different research centers.

Different reviews of the literature studied confirm that primary tumors that most metastasize in the oral cavity are those originating in breasts, lungs, kidneys and prostate (1,9,25). This review confirms that these origins are the most prevalent ones in terms of metastases in the oral region, but it should be noted that, as already discussed in previous studies, the nature of the primary tumor and the site of oral metastases have gender differences (1,26). In women, primary cancers that most metastasize in the oral cavity are those originating in breasts, followed by kidneys, lung, skin and colorectal neoplasms. On the other hand, the order of frequency changes in men, being lungs the most frequent neoplasms, followed by kidneys and much less frequently liver, soft tissues such as sarcoma or those of unknown origin (1,26).

Distant metastases are the result of hematogenous spread. However, Baranovic *et al*. (16) described a colorectal adenocarcinoma with metastases in the liver and oral cavity, but without affecting lungs or peritoneum, which are usually very common locations of metastases for this type of carcinomas. This can be explained by Batson's theory (16), which considers that there are 4 venous circuits: pulmonary, cava, portal and vertebral. In colorectal adenocarcinoma case, hematogenous dissemination through the vertebral circuit could occur, avoiding the neck and lungs region, thus producing oral metastases (16). It is considered that hard tissues metastases occur through hematogenous dissemination, being the mandible more frequently affected than the maxilla, probably due to the presence of terminal vascularization in the region and a greater presence of hematopoietic cells in the mandible, especially in the canines posterior region (8,21,27).

Clinical manifestations of oral metastases range from local pain or inflammation to paresthesia; therefore, its diagnosis is often complicated and delayed. They can be confused with pyogenic granulomas, giant cell granulomas, bone cysts, osteomyelitis or even with Paget's disease, among many other differential diagnoses (6,25,27). Many patients report jaw inflammation with localized pain (17).

No specific signs were reported in the studied cases: ulcerated, inflamed or painful oral lesions, with a tendency to bleeding or possible fistulization. Size, rapid growth, the tendency to bleed, necrotic appearance and/or the possibility of recurrence of the lesion, as well as the general condition of the patient will help in reaching a diagnosis and subsequently, through the histopathological study, will reach the final diagnosis (2,21,23).

Histopathology of the primary tumor, in most cases, resembles or mimics that of metastases in the oral cavity (8,17,25). Histological criteria for the diagnosis of a metastatic tumor are: a.- histopathology of the tumor must be verified primary, b.- metastatic tumor must be of the same subtype as the primary tumor and also, c.-direct expansion due to continuity of the primary tumor must be excluded.

The prevalence of oral metastases has been linked to the incidence of primary cancers that appear in a certain population. There can be a lot of variation between different geographic areas according to the prevalence of a particular malignant tumor. For example, while in Spain breast cancer is the most prevalent among women, in India it is cancer of the uterine cervix. Data on primary tumors is more prevalent in a certain population having the possibility of causing metastases in the oral cavity. This will vary depending on the country from which a certain population has been studied (17). Moreover, variations in the incidence of the primary tumors locations may be due to several factors such as genetic mutations (4,8,25,28).

Metastases in the oral cavity can occur in hard tissues, but also in soft tissues. There are more cases of metastases in hard tissues than in soft ones reported in the literature, being the alveolar mucosa and the tongue the most common locations when soft tissues are involved. For instance, tongue is more frequently involved because it is an organ rich in blood vessels where cells from the primary tumor can embolize (1,2).

Time in between oral metastases diagnostic and patient’s death varies from months to years, although the median survival ranges from 1 to 5 years. Both Hirshberg *et al*. (6) and Van der Waal *et al*. (3) reported a mean survival of 6-7 months. In our review, of all the cases studied that end up in death, the mean survival is 9.81 months, with a range that varies from 3 weeks to 30 months. Only one case recovered 12 months after treatment and the rest are not specified.

## Conclusions

It is important for the doctor/dentist to take into account oral metastases in the differential diagnosis of lesions in the oral cavity because, despite the fact that these are rare cases, they are a sign of widespread neoplasm, conferring poor vital prognosis of the patient. The primary tumor sites that most metastasize to the oral cavity are the lung in both genders and breasts in females, closely followed by liver and kidneys also in both genders. Although the majority of metastases in the oral cavity include jaws and gums, any location of the oral mucosa may be involved.
